# Low-Dose Epidural Dexmedetomidine as an Adjuvant to Bupivacaine Versus Conventional Bupivacaine for Postoperative Analgesia: A Randomized Controlled Trial

**DOI:** 10.7759/cureus.92018

**Published:** 2025-09-10

**Authors:** Nayana Sabu, Sucheta Meshram, Gajanan Chavan

**Affiliations:** 1 Department of Anaesthesiology and Critical Care, All India Institute of Medical Sciences, Nagpur, Nagpur, IND; 2 Department of Anaesthesiology and Critical Care, Jawaharlal Nehru Medical College, Wardha, IND

**Keywords:** analgesia, bupivacaine, dexmedetomidine, epidural, hemodynamics, postoperative

## Abstract

Background

Epidural analgesia is widely employed in postoperative care for abdominal and lower limb surgeries, offering superior pain control, reduced opioid consumption, and improved postoperative recovery compared to systemic opioids.

Objective

The objective of this study is to evaluate postoperative analgesia following lower limb and abdominal procedures using low-dose epidural dexmedetomidine with 0.0625% bupivacaine against 0.125% bupivacaine.

Methods

Two groups of sixty American Society of Anesthesiologists (ASA) grades I and II adults undergoing elective surgery were randomly assigned: Group BB received bupivacaine 0.125%, while Group BD received epidural bupivacaine 0.0625% with dexmedetomidine (0.5 µg/kg). Over a day, assessments were made of the visual analog scale (VAS), hemodynamics, motor block (Bromage scale), Richmond Agitation-Sedation Scale (RASS), and rescue analgesia.

Results

Group BD required fewer rescue analgesics and had substantially lower VAS scores (p = 0.003). In the BD group without clinical hypotension, heart rate and mean arterial pressure (MAP) were somewhat lower. There was no discernible change in motor block. There were a few and controllable side effects.

Conclusion

The addition of dexmedetomidine to epidural bupivacaine has been shown to provide superior analgesia, enhanced hemodynamic stability, and a reduced incidence of adverse effects when compared to bupivacaine alone.

## Introduction

Epidural analgesia is widely employed in postoperative care for abdominal and lower limb surgeries, offering superior pain control, reduced opioid consumption, and improved postoperative recovery compared to systemic opioids [1]. Epidural analgesia has been the gold standard used in major abdominal and lower-limb surgeries due to its beneficial pain control and reduction of the surgical stress response [2]. Bupivacaine is the most common drug used in epidural infusion; however, its drawbacks are dose-dependent motor blockade and changes in hemodynamics [3].

Dexmedetomidine is a highly selective agonist of α2-adrenergic receptors that has sedative, analgesic, and sympatholytic effects with minimal respiratory depression, and is an appealing epidural adjuvant [4,5]. Dexmedetomidine combined with bupivacaine has a synergistic effect, which allows the use of reduced concentrations of local anesthetics, augmenting analgesia and diminishing the side effects of profound motor block and hypotension [6,7]. Randomized trials and meta-analyses also prove that bupivacaine-dexmedetomidine combination provides better postoperative analgesia, hemodynamic stability, less use of opioids, and better motor-block properties than bupivacaine alone [8].

This research aims to compare the outcomes of conventional-dose bupivacaine and the low-concentration bupivacaine supplemented with dexmedetomidine on the postoperative pain levels, hemodynamic stability, motor block, and the overall quality of analgesia.

Aim

This study aims to compare conventional-dose epidural bupivacaine with low-dose bupivacaine plus dexmedetomidine for assessing postoperative analgesia, motor block, hemodynamic stability, and adverse effects.

Objectives

The primary objective of this study was to assess the quality of postoperative analgesia using the visual analog scale (VAS).

The secondary objective was to evaluate hemodynamic stability, defined as the absence of >20% deviation from baseline heart rate, mean arterial pressure (MAP), respiratory rate (RR), or pulse oximetry by trend analysis over time, incidence of motor block using the modified Bromage scale, monitor the incidence of any adverse effects, and record total consumption and timing of rescue analgesics.

## Materials and methods

Participants and study design

This trial was a randomized controlled and open-label trial. Over the course of one and a half years, from May 2024 to January 2025, sixty patients were enrolled. The Institutional Ethics Committee granted ethical approval (IEC/Pharmac/2024/728), and informed and written consent was taken from all participants.

Inclusion and exclusion criteria

The study included adult patients (18-60 years old) undergoing lower limb and abdominal procedures with American Society of Anesthesiologists (ASA) grades I and II under combined spinal and epidural anesthesia, who were included in the study population [9]. Patients were excluded if they had contraindications to regional anesthesia, were pregnant, exhibited altered sensorium, or experienced intraoperative hemodynamic instability. Additional exclusion criteria included pre-existing bradycardia (heart rate < 60 beats per minute (bpm)) and hypotension, defined as a MAP reduction of more than 20% from baseline.

Calculation of sample size

The sample size was calculated using the formula for comparing two means [3], with a power of 80% and a two-sided confidence interval (CI) of 95%. Based on the 48-hour postoperative VAS-R scores, the following parameters were used: the mean scores were 0.77 for Group 1 and 1.30 for Group 2, with standard deviations of 0.57 and 0.75, respectively, and a mean difference of 0.53.

This yielded a maximum calculated sample size of n = 29 per group. However, a minimum of 25 patients per group was determined to be statistically sufficient [3].

Methodology

Patients were randomly divided into two groups postoperatively in the Post-Anesthesia Care Unit (PACU):

Group BD (Bupivacaine + Dexmedetomidine)

Bupivacaine (0.0625%) + dexmedetomidine (0.5 mcg/kg) was administered via epidural infusion; 6.25 mL of 0.5% bupivacaine hydrochloride (ANAWIN), 0.5 mL of dexmedetomidine (50 mcg/cc, DEXTOMID), and 43.25 mL of 0.9% normal saline were combined to create the infusion. For 24 hours, the infusion rate was maintained at 3-7 mL/hour, titrated for hemodynamic stability and pain alleviation. The lead investigator paid for the dexmedetomidine. Initially, the epidural infusion was started at a rate of 5 mL/hour, titrated upward up to a maximum of 7 mL/hour if dermatomal coverage seemed insufficient, always within the 5-7 mL/hour window.

Group BB (Bupivacaine Only)

Participants received an epidural infusion of Bupivacaine (0.125%). The infusion was prepared by mixing 12.5 mL of 0.5% bupivacaine hydrochloride (ANAWIN) with 37.5 mL of 0.9% normal saline. The infusion rate was 3-7 mL/hour, titrated for pain relief and hemodynamic stability, and continued for 24 hours.

Epidural catheters were inserted in the operating theatre before general anesthesia. The level for epidural insertion was taken as L2-L3, preferably for abdominal and hip surgeries, and L3-L4 for surgeries of the knee, for better dermatomal coverage. Both drugs were stored at room temperature and were from the same batch. Study drugs were provided free of cost to the patients.

Randomization process

With a minimum sample size of 25 patients per group, randomization was performed using block randomization. The randomization sequence was generated via Microsoft Excel’s (Microsoft® Corp., Redmond, WA) random number function, and allocation was conducted using the sealed opaque envelope method. This process was carried out by a departmental staff anesthesiologist who was not part of the study team, to ensure allocation concealment and reduce bias.

Monitoring and assessment

Demographic Details

Age, ASA grade, operative time, and type of surgery were recorded.

Respiratory and hemodynamic parameters are tracked and recorded at 0, 2, 6, 12, 16, and 24 hours. Richmond Agitation-Sedation Scale (RASS), RR, MAP, pulse rate (PR), and peripheral capillary oxygen saturation (SpO2) were among the parameters that were recorded [10].

Quality of Analgesia

Analgesic quality was assessed using the VAS scale at rest at 0, 2, 6, 12, 16, and 24 hours [11]. VAS at movement was assessed at 12, 16, and 24 hours.

Motor Block

Motor block was assessed using the modified Bromage scale at 0, 2, 6, 12, and 24 hours [12].

Adverse Effects and Management

By standard procedures, potential adverse effects (such as hypotension, dizziness, bradycardia, nausea, vomiting, pruritus, shivering, urine retention, respiratory depression, and sedation) were tracked and treated.

Rescue analgesics (Inj. Tramadol 2 mg/kg IV, fentanyl patch 0.5 mcg/kg/hour for Group BD (The rescue dose of fentanyl patch is 0.5 mcg/kg for both study groups); Inj. Tramadol 2 mg/kg IV, fentanyl patch 25 mcg/kg/hour for Group BB) were provided in a stepwise approach.

Ethical considerations

This clinical trial was prospectively registered with the Clinical Trials Registry India (CTRI) under the registration number CTRI/2024/05/068224, in accordance with the ethical standards and regulatory requirements for human research.

## Results

**Figure 1:**
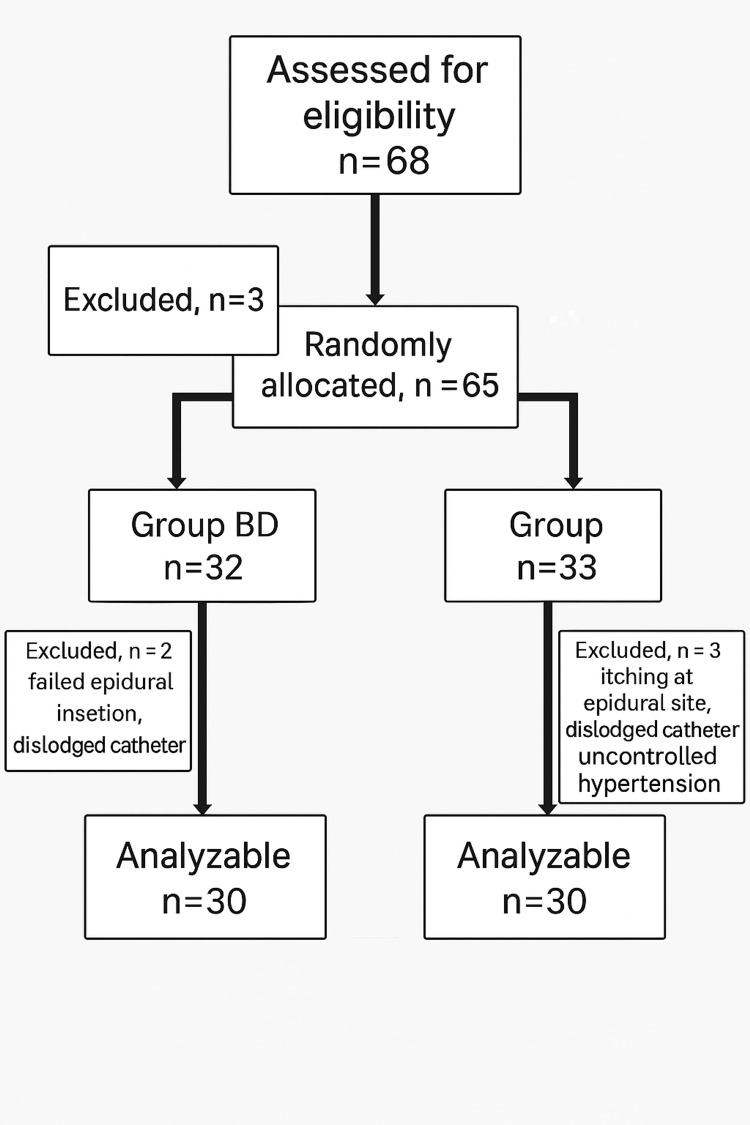
Flow diagram of patients This flowchart outlines the progression of participants through each stage of the randomized controlled trial. It shows the total number of patients assessed, randomized, excluded, and ultimately analyzed in the BD (bupivacaine + dexmedetomidine) and BB (bupivacaine only) groups.

Sixty-eight patients were screened, with 65 randomized: 32 in Group BD (bupivacaine + dexmedetomidine) and 33 in Group BB (bupivacaine only). After exclusions (two in BD, three in BB), each group included 30 patients for analysis, ensuring balanced cohorts post-dropout. Baseline characteristics, including age, ASA physical status, operative duration, and distribution of surgical procedures (total abdominal hysterectomy (TAH), ovarian cystectomy, total hip replacement (THR), total knee replacement (TKR), intertrochanteric (IT) fracture, staging laparotomy), were comparable across groups. The participant flow through the study, including screening, allocation, and analysis, is illustrated in Figure 1, confirming balanced group sizes post-exclusion.

Table 1 summarizes demographic and surgical parameters, such as age, ASA grade, operative time, and type of surgery, demonstrating well-balanced baseline characteristics across both groups. Cardiovascular parameters showed a significant heart-rate reduction of approximately 6 bpm in Group BD (bupivacaine + dexmedetomidine), with a 95 % CI of -7.88 to -4.13 (p < 0.001) across generalized estimating equation (GEE), generalized linear model (GLM), and mixed-effects analyses. MAP decreased modestly in BD (mean drop ~1.48 mmHg; 95% CI: -2.94 to -0.02; p = 0.046-0.047).

**Table 1:** Baseline demographic and surgical characteristics Demographic data, including patient and surgical factors. This table includes participant age, ASA classification, operative duration, and surgical type. Groups BD and BB were demographically well-matched, minimizing selection bias. ASA: American Society of Anesthesiologists; IT: intertrochanteric fracture; TAH: total abdominal hysterectomy; THR: total hip replacement; TKR: total knee replacement

Variable	Bupivacaine + Dexmedetomidine (n = 30)	Bupivacaine (n = 30)	p-value
Age (years)	47.13 ± 10.06	50.66 ± 8.74	0.152
ASA grade I/II	1.23 ± 0.43	1.34 ± 0.48	0.354
Operative time (minutes)	131.13 ± 33.10	143.97 ± 43.29	0.202
TAH	9	13	0.422
Ovarian cystectomy	9	7	0.770
THR	2	3	1.000
TKR	8	6	0.760
IT fracture	0	1	1.000
Staging laparotomy	2	0	0.492

Group BD (epidural bupivacaine 0.0625 % + dexmedetomidine 0.5 µg kg^-^¹) exhibited a significantly lower PR than Group BB (epidural bupivacaine 0.125 % alone). Model coefficients represent the mean between-group difference in heart rate (bpm); negative values favor Group BD (Table 2). Estimates were generated with three approaches: GEE, GLM, and mixed-effects regression, and are presented with their standard error, 95% CI, and two-sided p-value.

**Table 2:** Data analysis of heart rate in the intervention versus the control group Mean heart rate differences were analyzed using GEE, GLM, and mixed effects regression. Negative coefficients favor Group BD. All models showed statistically significant reductions in heart rate in the BD group (p < 0.001). bpm: beats per minute; CI: confidence interval; GEE: generalized estimating equation; GLM: generalized linear model

Model	Coefficient	Standard Error	95% CI Lower	95% CI Upper	p-value
GEE	-6.01	1.82	-9.56	-2.45	0.001
GLM	-6.01	0.96	-7.88	-4.13	0.000
Mixed effects	-6.01	0.96	-7.88	-4.13	0.000

**Figure 2:**
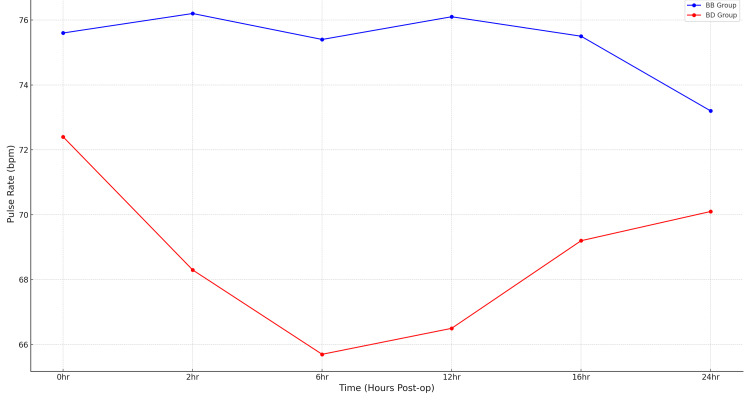
Trend of heart rate in both groups over 24 hours This figure compares heart rate changes at 0, 2, 6, 12, 16, and 24 hours postoperatively. Group BD consistently showed lower heart rates, reflecting the sympatholytic effect of dexmedetomidine. Data are expressed as mean ± standard error. BB: bupivacaine-only group; BD: bupivacaine + dexmedetomidine group; bpm: beats per minute

Line plot (Figure 2) of mean heart rate (bpm) at 0, 2, 6, 12, 16, and 24 hours for Group BD versus Group BB. The sustained reduction in Group BD reflects the sympatholytic action of dexmedetomidine, whereas Group BB maintains higher values. Error bars indicate ± standard error of the mean. Comparing postoperative analgesia in both groups, we found that BD patients had significantly reduced VAS pain scores (regression coefficient = -0.21;95% CI: -0.35 to -0.07; p = 0.003). Motor block assessment, evaluated using the Bromage scale, showed a mean score of 0.58 ± 1.03, with scores ranging from 0 to 3. As with VAS, Poisson models were employed due to the ordinal and skewed nature of the data. The comparison between groups yielded a regression coefficient of -0.14, with a 95% CI extending from -0.51 to 0.22. The associated p-value was 0.444, indicating no statistically significant difference in motor block scores between the intervention and control groups. Table 3 summarizes VAS pain scores over the first 24 hours. Negative coefficients indicate lower pain scores in Group BD compared with Group BB. Results were obtained using GEE (Poisson link), GLM (Poisson), and mixed-effects Poisson regression, and are displayed with standard error, 95 % CI, and p-values.

**Table 3:** Data analysis of the visual analog scale in the intervention versus the control group VAS scores were analyzed using Poisson regression across three models. Group BD had significantly lower pain scores (p = 0.003), confirming improved analgesic outcomes with dexmedetomidine.

Model	Coefficient	Standard Error	95% CI Lower	95% CI Upper	p-value
GEE (Poisson)	-0.21	0.082	-0.37	-0.05	0.011
GLM (Poisson)	-0.21	0.070	-0.35	-0.07	0.003
Mixed Poisson	-0.21	0.070	-0.35	-0.07	0.003

**Figure 3:**
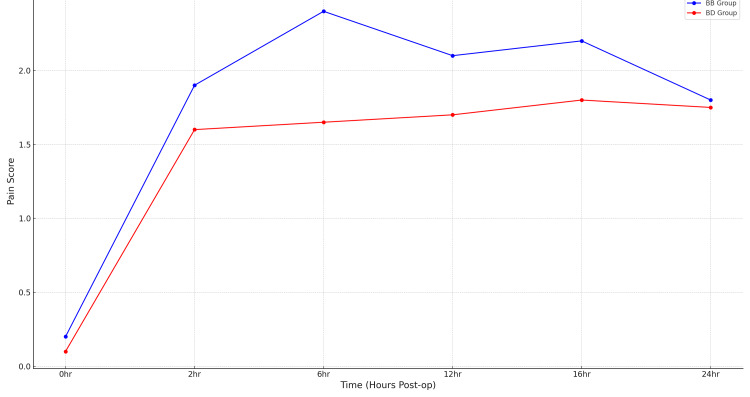
Trend of the visual analog scale in both groups over 24 hours Pain intensity at rest and during movement was recorded at several time points. Group BD demonstrated consistently lower pain scores compared to Group BB, indicating superior analgesic efficacy. Values range from 0 (no pain) to 10 (worst imaginable pain). BB: bupivacaine-only group; BD: bupivacaine + dexmedetomidine group; VAS: visual analog scale

Figure 3 illustrates the median VAS scores (0 = no pain, 10 = worst imaginable pain) at the same postoperative time points for Group BD and Group BB. Lower values across all intervals in Group BD confirm the superior analgesic efficacy of dexmedetomidine-enhanced epidural infusion. VAS scale at rest at 0, 2, 6, 12, 16, and 24 hours. VAS at movement was assessed at 12, 16, and 24 hours. VAS scores were analyzed using Poisson regression across three models. Group BD had significantly lower pain scores (p = 0.003), confirming improved analgesic outcomes with dexmedetomidine (Figure 3 and Table 3).

In the BD group, only two out of 15 patients (13.3%) required rescue analgesia. In the BB group, three out of 15 patients (20%) required rescue analgesia. In the BD group, only two out of 30 patients (6.6%) required rescue analgesia. In the BB group, three out of 30 patients (10%) required rescue analgesia. Comparing the time to breakthrough pain, the mean time to breakthrough pain for patients requiring rescue analgesia in the BD group was approximately 10 hours post-surgery. In contrast, in the BB group, breakthrough pain occurred significantly earlier, with an average time of around six hours post-surgery.

One BD patient experienced transient motor weakness at hour 6; one BB patient had mild bradycardia responsive to glycopyrrolate. Respiratory parameters remained stable across groups.

Motor block assessment, evaluated using the Bromage scale, showed a mean score of 0.58 ± 1.03, with scores ranging from 0 to 3. As with VAS, Poisson models were employed due to the ordinal and skewed nature of the data. The comparison between groups yielded a regression coefficient of -0.14, with a 95% CI extending from -0.51 to 0.22. The associated p-value was 0.444, indicating no statistically significant difference in motor block scores between the intervention and control groups.

## Discussion

An essential part of multimodal surgical care is postoperative analgesia. It lowers opioid-related adverse effects, improves patient satisfaction, eases early mobilization, and lessens surgical stress. Because of its focused action and opioid-sparing effect, epidural analgesia is still very useful in lower abdomen and limb procedures. Dexmedetomidine, a highly selective α2-adrenergic agonist, has the best profile of any adjuvant available since it has analgesic, sedative, and sympatholytic actions without causing any respiratory depression or motor impairment. In this randomized, double-blind trial, 60 patients were equally allocated to receive either bupivacaine with dexmedetomidine (Group BD) or bupivacaine alone (Group BB). Both groups were well-matched in age, ASA physical status, gender distribution, and surgical type. This demographic consistency reduces confounding and supports internal validity. A similar demographic balance was strengthening the external generalizability of our findings [1,2]. Hetta et al. also emphasized the methodological soundness of such demographic parity, despite including a higher-risk and older patient cohort [3].

**Figure 4:**
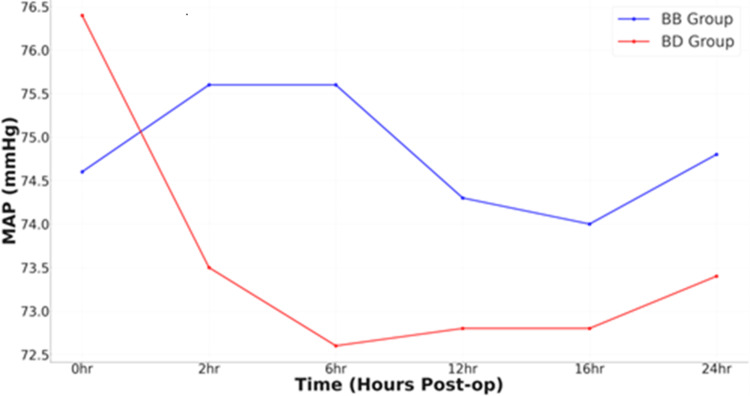
Trend of mean arterial pressure in BB (control group) versus BD (intervention group)

Figure 4 shows the trend of MAP in the BB (control group) versus the BD (intervention group). Table 4 illustrates the data analysis of MAP in the BB (control group) versus the BD (intervention group).

**Table 4:** Data analysis of mean arterial pressure (MAP) in BB (control group) versus BD (intervention group) GEE: generalized estimating equation; GLM: generalized linear model

Model	Coefficient	Standard Error	95% CI Lower	95% CI Upper	p-value
GEE	-1.48	1.39	-4.2	1.23	0.285
GLM	-1.48	0.75	-2.94	-0.02	0.046
Mixed effects	-1.48	0.75	-2.95	-0.02	0.047

The BD group experienced a significant decrease in heart rate (p < 0.001), which is in line with the established sympatholytic action of dexmedetomidine through presynaptic norepinephrine suppression. While MAP decreased slightly, it remained within a clinically acceptable range. Jain et al. and Zhao et al. also demonstrated decreased heart rates and MAP without clinical instability [4,5]. Meta-analytic evidence by Zhang et al. supports our findings, noting no statistically significant risk of hypotension or bradycardia [6]. Gong et al. further highlighted dexmedetomidine’s cardioprotective role during cardiac surgery [7]. Aho et al. observed reduced BP variability and isoflurane needs with dexmedetomidine, although without HR changes [8]. The BD group exhibited significantly lower VAS pain scores (p = 0.003), longer pain-free intervals (10 versus six hours), and fewer requirements for rescue analgesia (13.3% versus 20%). This affirms dexmedetomidine’s analgesic superiority and opioid-sparing benefits. Hetta et al. and Patil et al. reported similar findings with reduced opioid consumption and prolonged block durations [3,13]. El Kabarity et al. [14] and R S et al. demonstrated enhanced analgesic efficacy and satisfaction in laboring women [15].

Modified Bromage scores between groups (p = 0.444), indicating that dexmedetomidine does not adversely impact motor function [12]. This characteristic is particularly valuable in enhanced recovery protocols. Kaur et al. showed that dexmedetomidine extended analgesia without impairing motor ability [16]. Techanivate et al. also found it beneficial in outpatient surgeries, enabling early ambulation and discharge [17]. Dexmedetomidine's high α2:α1 selectivity ensures potent sedation without respiratory compromise. Patients remained calm, cooperative, and easily arousable. This sedative profile supports safe postoperative monitoring and cognitive recovery. Priye et al. found similar results, with mild sedation and no hemodynamic compromise. They also observed a non-significant reduction in postoperative delirium [18].

Respiratory parameters remained stable across both groups. No cases of desaturation, hypoventilation, or respiratory depression occurred. Wahlander et al. reported comparable safety, noting improved ventilation and reduced opioid needs when dexmedetomidine was used in conjunction with epidural analgesia [19]. Table 5 shows a comparison of rescue analgesia requirement, mean time to breakthrough pain, and rescue medication between groups.

**Table 5:** Rescue analgesia use BB: bupivacaine-only group; BD: bupivacaine + dexmedetomidine group

Group	Patients Requiring Rescue Analgesia (n)	Mean Time to Breakthrough Pain (Hours) Rescue Analgesia Used
Group BD	2	10 Tramadol 2 mg/kg IV
Group BB	3	6 Tramadol 2 mg/kg IV

The BD group had a delayed time to initial analgesic request and a lower demand for rescue analgesia. Wahlander et al. supported these findings, documenting reduced fentanyl requirements without affecting pain scores [19]. Gandhi et al. and Manne et al. observed stable hemodynamic and respiratory profiles in peripheral and general anesthesia settings [20,21]. Coskuner et al. highlighted additional benefits, including reduced postoperative shivering and manageable bradycardia [22].

A wide range of literature reinforces our findings. Li et al. concluded that dexmedetomidine provides superior analgesia over placebo and equivalent outcomes to opioids with fewer adverse effects [23]. Azemati et al. found extended block durations with dexmedetomidine compared to meperidine [24]. Qian et al. emphasized improved analgesic onset and duration with reduced nausea and vomiting [2,25]. Zhang et al. and Lao et al. demonstrated that adding dexmedetomidine as an adjuvant in intrathecal labor analgesia improved analgesic efficacy and maternal satisfaction, with an acceptable safety profile [6,26]. Lu et al. demonstrated that dexmedetomidine reduced oxidative stress and protected distal organ function after lower limb ischemia-reperfusion in elderly patients undergoing knee arthroplasty, highlighting an added benefit of the drug beyond its established roles in providing analgesia and maintaining hemodynamic stability [27]. A small sample size and the inclusion of primarily ASA I-II patients are two limitations of the current study that restrict its applicability to high-risk or geriatric populations. The exclusion of pediatric and geriatric subgroups, as well as the absence of long-term follow-up for chronic pain and functional outcomes, are further limitations [28-30].

Large-scale multicenter trials to confirm these results across larger populations, such as ASA III-IV, the elderly, and pediatric groups, should be part of future directions. Comparative studies with other non-opioid adjuvants like clonidine, nalbuphine, or magnesium sulfate are needed to determine relative efficacy and safety. Our study highlights the need for future research exploring dexmedetomidine analogs with improved safety, building on preclinical structure-activity relationship (SAR) findings to develop next-generation α2-agonists for safer, more effective perioperative analgesia.

Our study demonstrated that dexmedetomidine, when used as an adjunct to a lower dose of bupivacaine, exhibited drug synergism by enhancing analgesia, permitting local anesthetic dose reduction, and thereby minimizing the side effects associated with conventional bupivacaine dosing.

## Conclusions

This randomized controlled trial has shown that including a low dose of dexmedetomidine with epidural bupivacaine produces better analgesic results in the post-surgery stage than the traditional bupivacaine alone. Patients in the BD group reported lower pain scores, decreased need for rescue analgesics, and an even longer pain-free period, all without an increase in motor blockade or compromised respiration. Although hemodynamic parameters showed only modest effects, they remained within clinically acceptable ranges, indicating the safety and tolerability of the combination. Notably, the sedative effects of dexmedetomidine were minimal, and none of the patients’ cooperation or recovery was affected. The results align with other studies highlighting dexmedetomidine’s analgesic and opioid-sparing properties, supporting its use in enhanced recovery protocols. However, the limited sample size and exclusion of high-risk populations restrict the extent to which the study can influence the integration of dexmedetomidine into multimodal pain management strategies. Nonetheless, it provides strong evidence for its potential. Future research should include more diverse groups and compare dexmedetomidine with other non-opioid adjuvants to verify replicability. Low-dose dexmedetomidine combined with epidural bupivacaine provides superior analgesia with stable safety profiles, supporting its role as an effective adjuvant in multimodal postoperative pain management.
